# All-Optical Domain Inversion in LiNbO_3_ Crystals by Visible Continuous-Wave Laser Irradiation

**DOI:** 10.1021/acsphotonics.4c00336

**Published:** 2024-06-21

**Authors:** Carlos Sebastián-Vicente, Jörg Imbrock, Simon Laubrock, Olga Caballero-Calero, Angel García-Cabañes, Mercedes Carrascosa

**Affiliations:** † Departamento de Física de Materiales, Universidad Autónoma de Madrid, 28049 Madrid, Spain; ‡ Instituto Nicolás Cabrera, Universidad Autónoma de Madrid, 28049 Madrid, Spain; § Institute of Applied Physics, University of Münster, Corrensstr. 2, 48149 Münster, Germany; ∥ Instituto de Micro y Nanotecnología, IMN-CNM, CSIC (CEI UAM+CSIC) Isaac Newton, 8, Tres Cantos, E-28760 Madrid, Spain

**Keywords:** lithium niobate, ferroelectric materials, domain inversion, laser processing, bulk photovoltaic effect, interfacial screening

## Abstract

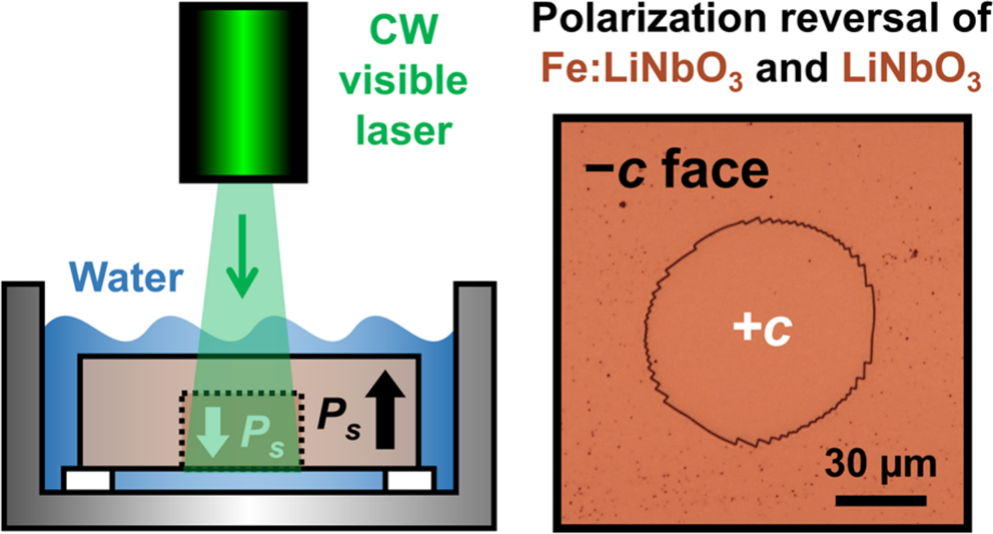

LiNbO_3_ is a distinguished multifunctional material where ferroelectric domain engineering is of paramount importance. This degree of freedom of the spontaneous polarization remarkably enhances the applicability of LiNbO_3_, for instance, in photonics. In this work, we report the first method for all-optical domain inversion of LiNbO_3_ crystals using continuous-wave visible light. While we focus mainly on iron-doped LiNbO_3_, the applicability of the method is also showcased in undoped congruent LiNbO_3_. The technique is simple, cheap, and readily accessible. It relies on ubiquitous elements: a light source with low/moderate intensity, basic optics, and a conductive surrounding medium, e.g., water. Light-induced domain inversion is unequivocally demonstrated and characterized by combination of several experimental techniques: selective chemical etching, surface topography profilometry, pyroelectric trapping of charged microparticles, scanning electron microscopy, and 3D Čerenkov microscopy. The influence of light intensity, exposure time, laser spot size, and surrounding medium is thoroughly studied. To explain all-optical domain inversion, we propose a novel physical mechanism based on an anomalous interplay between the bulk photovoltaic effect and external electrostatic screening. Overall, our all-optical method offers straightforward implementation of LiNbO_3_ ferroelectric domain engineering, potentially sparking new research endeavors aimed at novel optoelectronic applications of photovoltaic LiNbO_3_ platforms.

## Introduction

Over many decades, lithium niobate (LN) has been a major playground for photonics research due to the rich variety of properties combined in a single-material platform. LN stands out for its high electrooptic coefficients with fast response, high nonlinear coefficients for second-order χ^(2)^ and third-order χ^(3)^ processes, strong photorefraction, and broad transparency window (350–5500 nm).^1^ These remarkable properties make it possible to tailor this platform for a number of widespread photonic applications: electrooptic modulators,^2^ integrated spectrometers,^3^ hologram storage,^4^ frequency conversion,^5−7^ frequency comb generation,^8,9^ optofluidic lab-on-a-chip devices,^10,11^ or various nonlinear nanophotonic applications,^12^ among others. Most of the aforementioned applications of LN are ultimately enabled by its noncentrosymmetric and ferroelectric nature. In fact, LN has a remarkably high Curie temperature of 1210 °C, showing a ferroelectric polar phase in a broad temperature range. Due to the lack of inversion symmetry of the lattice in the polar phase, LN also exhibits the piezoelectric, pyroelectric, and bulk photovoltaic (PV) effects, which are particularly strong in this material.^13^ This provides even more degrees of freedom to LN, both inside and outside the realm of photonics.

In the framework of ferroelectricity, some of the applications of LN require, or can benefit from, customized ferroelectric domain structures.^14^ The most classical example is frequency conversion by second-harmonic generation (SHG), where periodically poled structures are needed to achieve quasi-phase matching.^5^ However, domain-engineered structures in LN are also of great interest for beam steering,^15^ surface acoustic wave devices,^16^ conductive domain walls for nanoelectronics,^17^ trapping and patterning of particles,^12,18−20^ and many more.

The traditional method for domain inversion consists in the application of external electric fields above the coercive field (∼210 kV cm^–1^ in congruent undoped LN) in the opposite direction of the spontaneous polarization.^14,21^ A high-voltage supply must be used to overcome the coercive threshold of LN. Currently, lithography-based electrical poling offers the best performance to fabricate periodically poled LN structures, enabling superior throughput for large-area devices (>1 cm^2^) with spatial resolution in the micrometer scale and up to millimeter domain depths. Another key advantage of this standard method is that it can be applied to doped and undoped LN, as well as other ferroelectrics. However, to achieve the desired domain patterns, electrodes are patterned on the crystal by lithography techniques, making the process cumbersome and costly. A clean room is needed and a custom mask is required for each specific application. Moreover, the fabrication of domain structures with submicrometer resolution is hindered by the lateral growth of the inverted domains beyond the extent of the patterned electrodes. In addition, there exist other electrical poling techniques with some advantages over lithography-based poling. These methods do not require clean rooms, masks, or complex photolithography steps. On the one hand, using an externally biased tip of a scanning probe microscope, micro- and nanoscale domain structures can be fabricated.^22^ However, an atomic force microscope is needed, the generation of large-area devices is impractical, and domain structures are shallower compared to lithography-based poling. On the other hand, the so-called calligraphic poling employs a scanning microtip electrode instead of an atomic force microscope, making the equipment significantly simpler and cheaper.^23,24^ Although the writing speed is slower than lithography-based poling, large-area domain structures can be tackled (>1 cm^2^), with domain sizes in the micrometer range and depths similar to those of lithographic electrical poling.

As an alternative, other domain inversion methods have been proposed, such as electron beam irradiation,^25^ ion beam irradiation,^26^ or light-mediated poling.^27^ While electron/ion beams are capable of high resolutions, they require expensive and complex equipment, they are constrained to shallow domains, and they lack throughput for large-area patterning.^14^ However, certain light-based techniques are promising candidates that can overcome some of the limitations of these methods, taking advantage of the intrinsic patterning capabilities of light.

Optical domain engineering falls into two major groups: light-assisted poling and all-optical poling.^27^ While all-optical techniques purely rely on light itself, light-assisted methods still require an external electric field^28−33^ or additional processing steps, such as film patterning by electron beam lithography^34^ or pyroelectric thermal treatments.^35,36^ All-optical methods completely circumvent the need for additional electric fields or processing steps. All-optical poling can be subclassified as a function of the excitation wavelength:

- UV light (pulsed or continuous-wave CW).^37−39^
- Far-IR light at λ = 10.6 μm (pulsed).^40^
- Near-IR light (pulsed).^41−45^

The first two methods share that they work outside the transparency window of LN, either with above-bandgap UV light or IR light beyond 5.5 μm. Thus, the absorption is remarkably high, leading to thermally induced electric fields due to local temperature variations. Different contributing mechanisms have been invoked in the literature, such as the pyroelectric effect, Li diffusion, or the thermoelectric effect.^27,39^ In turn, due to the strong absorption, the penetration depth is low, leading to shallow domain structures. Conversely, femtosecond near-IR lasers work within the transparency window of LN, overcoming this limitation. In fact, they have recently unlocked the possibility to realize 3D ferroelectric domain structures in bulk LN.^42,43^ This method relies on multiphoton absorption processes, hence requiring high intensities achieved by tight spatiotemporal light focusing. In this case, domain inversion has been attributed dominantly to the thermoelectric effect, induced by steep temperature gradients at the laser focus.^41^

Herein, we report the first method for all-optical domain inversion by CW visible light irradiation in LN crystals. Most of this work is focused on iron-doped LN (Fe:LN), where Fe impurities introduce an absorption band in the visible spectrum. Despite this absorption band, Fe:LN crystals are partially transparent in the visible range at usual doping levels, allowing for deep penetration of light, similarly to methods based on near-IR light. Moreover, it is widely known that iron dopants boost the bulk PV effect of LN, leading to the highest saturation electric fields of all PV materials to date (up to ∼200 kV cm^–1^).^46,47^ While Fe:LN has been very well known and studied for many years in holography, only recently has it emerged as an outstanding optoelectronic platform for the light-driven actuation on different systems via its large electric fields. Indeed, the ability to generate customized electric field distributions by structured light, without external power supplies or lithography-patterned electrodes, makes Fe:LN a rather versatile and appealing asset. For example, Fe:LN has been successfully employed for the optofluidic manipulation of liquid droplets,^48−53^ manipulation and patterning of micro/nanoparticles by PV optoelectronic tweezers,^54−58^ guided locomotion and alignment of liquid crystals,^59−62^ optical gating of graphene,^63^ or hybrid Fe:LN-graphene metasurfaces.^64^ All of these applications have been developed with monodomain Fe:LN crystals. However, multidomain structures could enhance the flexibility and potential of Fe:LN platforms, allowing the light-induced generation of arbitrary 2D bipolar charge distributions on *z*-cut substrates.

The method presented in this work offers an appealing scheme to easily tackle the flexible fabrication of such domain-engineered LN substrates. It consists in the light irradiation of the ferroelectric LN crystal while it is immersed in an electrically conductive medium, such as water. Thus, our all-optical method stands out for its extreme simplicity and low cost: it only requires a CW visible light source, basic optics, and a suitable liquid like water. These elements are ubiquitous, making the method straightforwardly accessible to anyone. No power supplies, lithography-patterned electrodes, or clean rooms are needed, as in conventional electric field poling. Another advantage is that low/moderate optical power densities on the order of W cm^–2^ are enough for all-optical domain inversion. Low-power visible lasers are easier to handle and align, safer, and more affordable compared to UV, femtosecond near-IR, or far-IR lasers.

First, in this manuscript, we provide a set of unequivocal tests that demonstrate all-optical domain inversion: selective chemical etching, surface profilometry, and pyroelectric trapping of charged microparticles. Then, we study the role of different parameters in the morphology of the inverted domains: exposure time, light intensity, focusing conditions, and surrounding medium. Furthermore, we characterize the 3D bulk structure of the domains by means of Čerenkov SHG microscopy. Remarkably, the deep penetration of light in the visible spectrum allows for domain structures deeper than any other all-optical technique without damaging the crystal (up to 289 ± 9 μm in this work). Finally, we demonstrate that the method is not constrained to Fe:LN, but can also be extended to undoped congruent LN. Based on the results derived from this comprehensive phenomenological characterization, we develop a novel qualitative model whereby light-induced ferroelectric switching is mainly driven by the bulk PV effect, assisted by interfacial screening charges.

## Experimental Procedure for All-Optical Domain Inversion

### Experimental Setup

**Figure 1:**
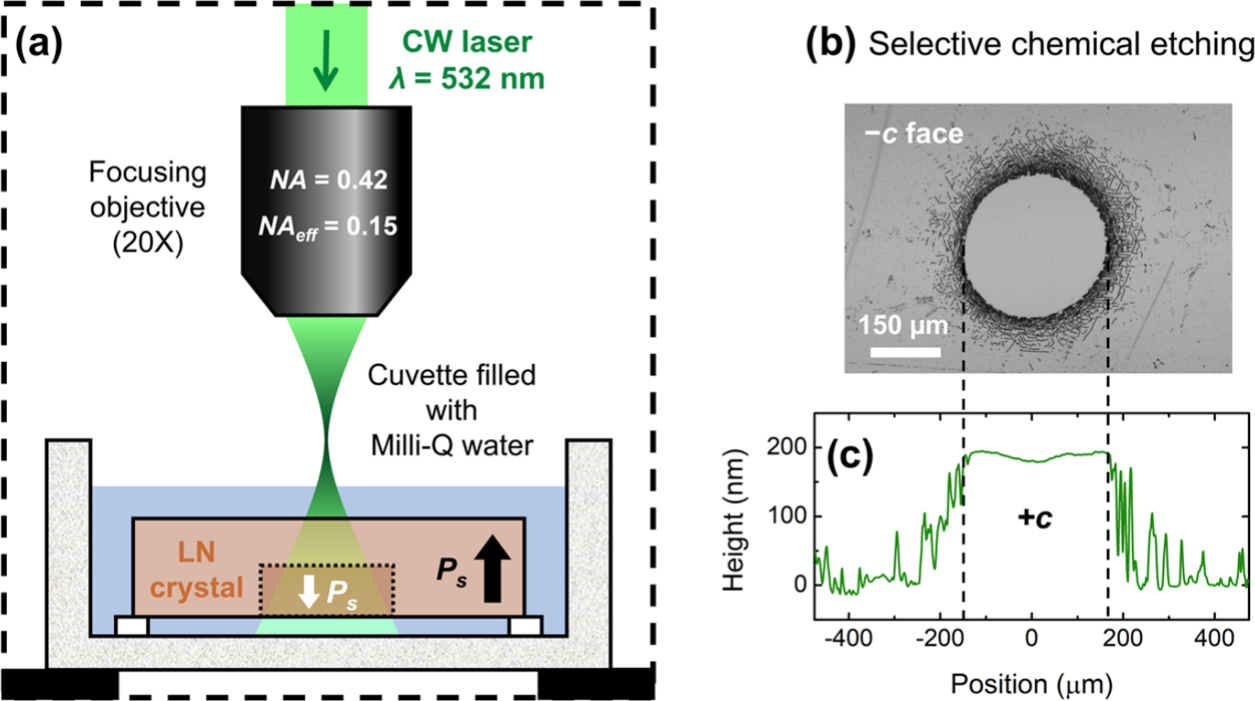
(a) Simplified schematic of the experimental setup for all-optical domain inversion. (b) Optical bright-field image of a laser-irradiated spot in Fe:LN (intensity *I* = 86.4 W cm^–2^, exposure time *t* = 1 min) after selective chemical etching with HF acid. (c) Measurement of the surface topography profile after selective chemical etching along the domain diameter.

As already mentioned, the experimental arrangement needed for all-optical domain inversion is strikingly simple. The basic setup employed in this work is illustrated in Figure 1a. Essentially, a CW laser operating at λ = 532 nm (model Coherent Verdi, maximum power of ≈1 W) is focused by an objective lens (magnification 20X, full numerical aperture NA = 0.42). Since the input laser does not cover the full pupil of the objective, the effective numerical aperture is NA_eff_ = 0.15. Then, the focused laser beam impinges on the LN sample, which is surrounded by ultrapure Milli-Q water inside a cuvette (unless otherwise specified). The cuvette is mounted on top of an XYZ stage. Unless otherwise stated, the crystal is located below the beam waist, as illustrated in Figure 1a. In this configuration, the default spot size of the Gaussian beam has a 1/e^2^ diameter of *d* = 4σ = 150 μm at the top surface and approximately 280 μm at the bottom, due to its divergence. In this definition, 2σ corresponds to the beam radius at which the intensity of the Gaussian profile drops to 1/e^2^ of its peak value at the center. The light intensities specified in the manuscript correspond to the average intensity of the Gaussian beam at the −*c* face (that is, half of the peak intensity). The intensity is calculated as *I* = *P*^–^/π(2σ)^2^, where *P*^–^ is the optical power at the −*c* face including absorption and reflection losses (see Section A in the Supporting Information for further details). This setup is embedded in a custom-made optical microscope (see Figure S1 in the Supporting Information).

In this work, we have predominantly used *z*-cut Fe:LN crystals, but also undoped congruent LN. Both are initially monodomain and have a thickness of 1 mm. The Fe:LN crystals have a 0.25 mol % iron concentration and an absorption coefficient of α_abs_ = 20.4 cm^–1^ at 532 nm. Prior to every experiment, the samples were carefully cleaned with distilled water and acetone, using lens cleaning tissue. During the experiments, the crystals were always oriented so that the incident face of the incoming laser beam was the +*c* face, as shown in Figure 1a.

### Demonstration of Light-Induced Domain Inversion

During light excitation of Fe:LN crystals surrounded by Milli-Q water, we observed in bright-field transmission microscopy that some material modifications were generated at the −*c* face, i.e., at the bottom nonincident face (see Video S1 in the Supporting Information). These modifications were stable and did not disappear over time after the experiment, even after the dark decay of the PV electric fields in a few days. Interestingly, nothing was observed at the +*c* face. Based on this preliminary hint, we hypothesized that ferroelectric domain inversion driven by light could be taking place. Thus, we carried out several tests.

To unequivocally reveal the light-induced inverted domains, the LN crystals were subjected to selective chemical etching in HF acid (purchased from J.T.Baker, 49% concentration) for 15 min at room temperature. This standard technique is based on the differential etch rate of the polar faces in LN: +*c* faces are essentially unaffected, whereas −*c* faces are appreciably etched.^65^ Both polar faces of the crystals were exposed to the acid during etching. A representative result is shown in Figure 1b, which clearly proves that domain inversion takes place at the −*c* face as a consequence of light excitation. Again, no inverted domains were observed at the +*c* face. Moreover, chemical etching provides insights into the surface domain morphology, which is discussed in detail in the next section.

Finally, we measured the surface topography profile after etching using a contact stylus profilometer (Dektak IIA). The height profile of Figure 1c indicates that the nonilluminated −*c* surface is etched faster than the illuminated (inverted) spot, as expected. This result is again consistent with the fact that +*c* domains are not appreciably affected by HF acid, while −*c* domains are. Moreover, from the height of the step (189 ± 18 nm), the differential etch rate can be estimated as 0.76 ± 0.07 μm h^–1^, in good agreement with the etching rate of the −*c* face reported in the literature for undoped LN in similar conditions.^65^ Additionally, we also exploited the pyroelectric effect to decorate the light-induced ferroelectric domains using charged microparticles (see Section C in the Supporting Information), confirming the reversal of the pyroelectric charge sign at the irradiated spot, consistent with the etching results. Therefore, the results of Figure 1 undoubtedly demonstrate all-optical domain inversion of Fe:LN crystals.

## All-Optical Domain Inversion in Fe:LiNbO_3_

In this section, the influence of the illumination parameters in the process of ferroelectric domain inversion is studied.

### Role of Exposure Time and Light Intensity

**Figure 2:**
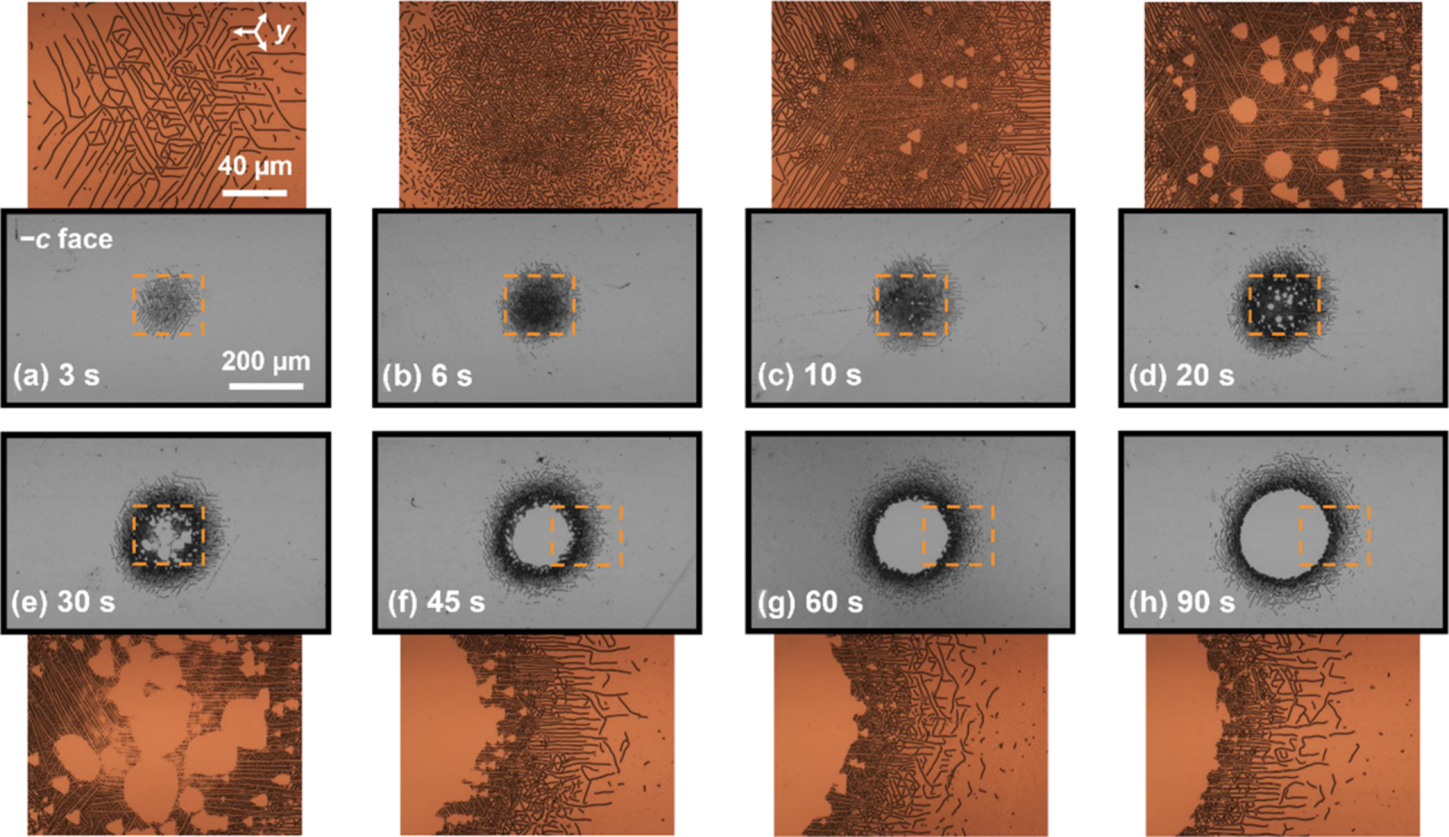
Optical bright-field images (after etching) of light-induced inverted domains for different exposure times from (a) 3 s up to (h) 90 s. In all cases, the light intensity is *I* = 28.8 W cm^–2^. The brown-colored images correspond to magnified areas of the wide-field grayscale images (indicated by a dashed rectangle). The scale bar of 200 μm is equal for all of the grayscale images, whereas the scale bar of 40 μm applies to all of the brown-colored magnified areas.

In Figure 2, eight experiments with different exposure times but fixed intensity are shown. Additional high-resolution images taken with a scanning electron microscope (SEM) may be found in Section D in the Supporting Information (Figures S4–S6). Clearly, the morphology of the inverted domains strongly depends on the exposure time. Based on these results, one can establish the following kinetics of the domain formation during light excitation:

(1) Nucleation starts in the form of sparse nanodomains in the illuminated area, as shown in Figure S5a for a lower intensity. Most of these submicron domains are irregular hexagons (quasi-triangles) with the vertices oriented in the *y* crystallographic directions.
(2) Then, the nanodomains serve as seeds for the growth of self-assembled nanoscale maze-like structures (see Figure 2a). These intricate structures are not random, but preferentially grow in the *y* directions (see also 2D Fourier transform in Figure S8). The typical width of the maze branches is in the range between ∼50 nm and about ∼1 μm. Also, the longer the exposure time, the thicker the branches and the more closely packed the maze becomes (see Figure 2b). The emergence of such a complex domain structure is thought to be due to correlated nucleation in the irradiated area.^14^
(3) When the density of domains in the maze is high enough, scattered triangular domains start to form (see Figure 2c), all of them oriented with the corners pointing in the +*y* crystallographic directions. The generation of triangular domains instead of hexagons is attributed to comparable switching and screening rates, as discussed in ref (14). In this case, the photoconductivity of Fe:LN can contribute to bulk charge redistribution after domain reversal, allowing domain stabilization, while the surrounding water contributes to surface screening. The observed triangles exhibit various sizes, and they grow over time (see Figure 2d). In some cases, one can see that the triangular shape is lost, evolving into rounded geometries. Eventually, coalescence occurs between the triangles themselves (see Figure 2e).
(4) In the end, complete coalescence takes place at the center of the illuminated spot (see Figure 2f), producing a fully inverted quasi-circular domain. Meanwhile, a halo of densely packed “tentacles” persists at the edge, inherited from the former maze. These tentacles have typical widths in the range between ∼50 nm and ∼1 μm (see Figure S4), and some of them have triangular protuberances with sizes up to ∼6 μm. Also, the orientation of the tentacle-shaped domains is not random: they point preferentially in the three crystallographic −*y* directions at different positions of the halo (see Figure S7).
(5) Over time, the fully inverted domain keeps growing sideways (see Figure 2g,h), increasing the diameter of the inner circle.

**Figure 3:**
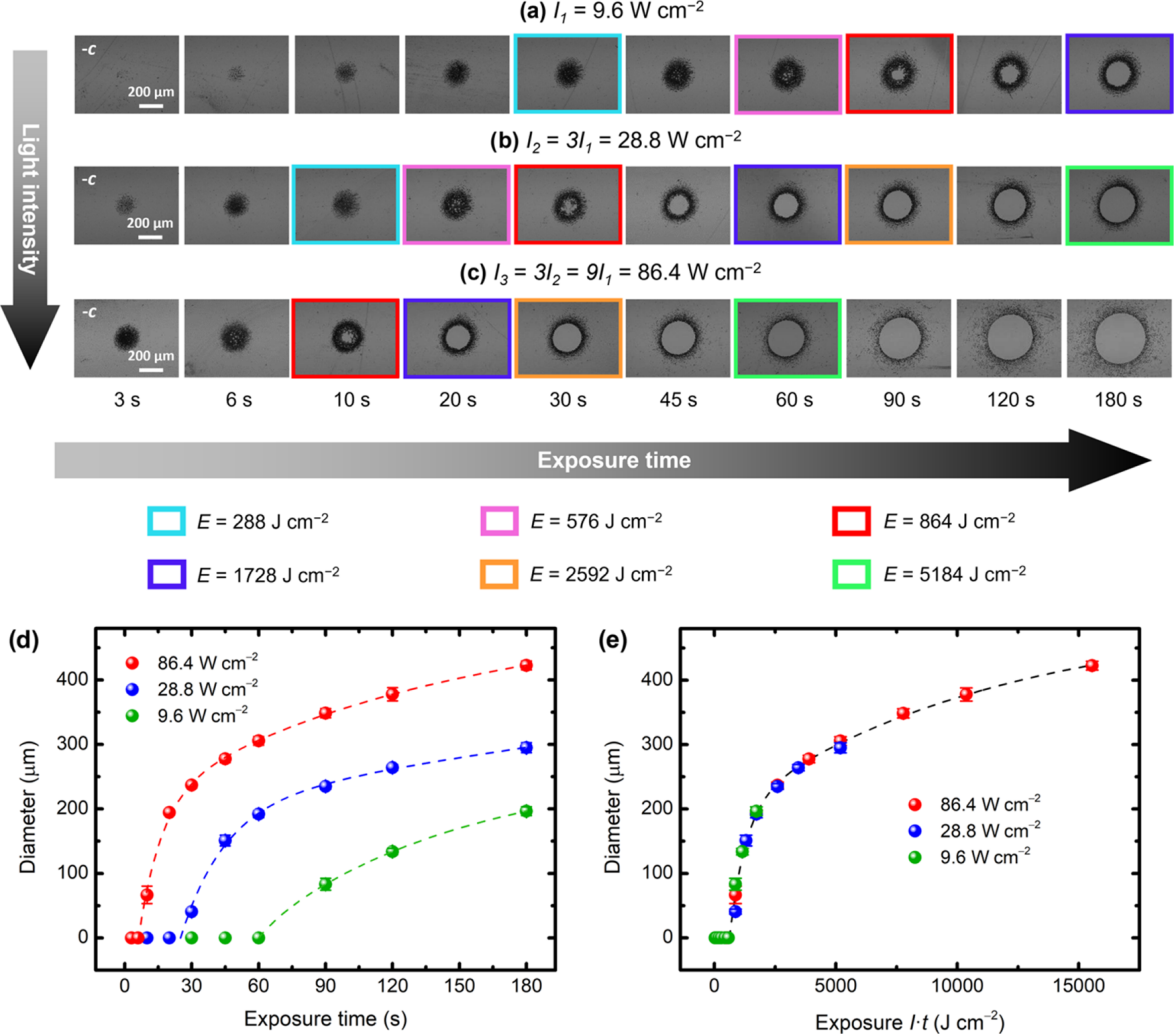
(a–c) Bright-field images of 30 domain inversion experiments using different light intensities and exposure times (after etching). Each row corresponds to a different light intensity (*I*), and each column corresponds to a different exposure time (*t*). The images with equally colored frames correspond to results with identical exposures (*E* = *I*·*t*). In the bottom graphs, the diameter of the inner circular domains is plotted as a function of (d) exposure time and (e) exposure. The dashed lines are fits to a function *f*(*x*) = *A*_1_·(1 – *e*^–(*x*–*x*_0_)/*x*_1_^) + *A*_2_·(1 – *e*^–(*x*–*x*_0_)/*x*_2_^), where *x* is either exposure time in (d) or exposure in (e). The fits are only included as a visual guide.

To shed more light on the role of the optical parameters, we conducted further experiments with different intensities and exposure times, as shown in Figure 3. The first observation from these results is that the kinetics of domain formation are faster for increasing light intensities (see also Video S2). Furthermore, the intensities and some of the exposure times were deliberately chosen so that they were integer multiples of each other, with a multiplication factor of 3. As a result, one can compare spots with different light intensities and exposure times, but equal exposure *E* = *I*·*t*. These cases are highlighted with frames of different colors in Figure 3a–c, where one can qualitatively see that such spots are at the same stage of the domain inversion process in terms of morphology. This surprising feature is also quantitatively demonstrated in Figure 3d,e. When the diameter of the inner inverted circle is plotted as a function of exposure (Figure 3e) instead of exposure time (Figure 3d), all of the curves merge into a single one. Notably, a time evolution governed by exposure is a well-known fingerprint of the bulk PV effect in the one-center model for Fe:LN.^66^ Moreover, a threshold exposure of about ∼620 J cm^–2^ can be inferred from Figure 3e for the formation of quasi-circular domains due to coalescence.

### 3D Characterization by Čerenkov SHG Microscopy

To gain insights into the depth of the light-inverted domains, we employed a Čerenkov SHG microscope, a widely used technique for 3D mapping of ferroelectric domains.^67,68^ Details about the setup and the measurements may be found in Section F in the Supporting Information.

**Figure 4:**
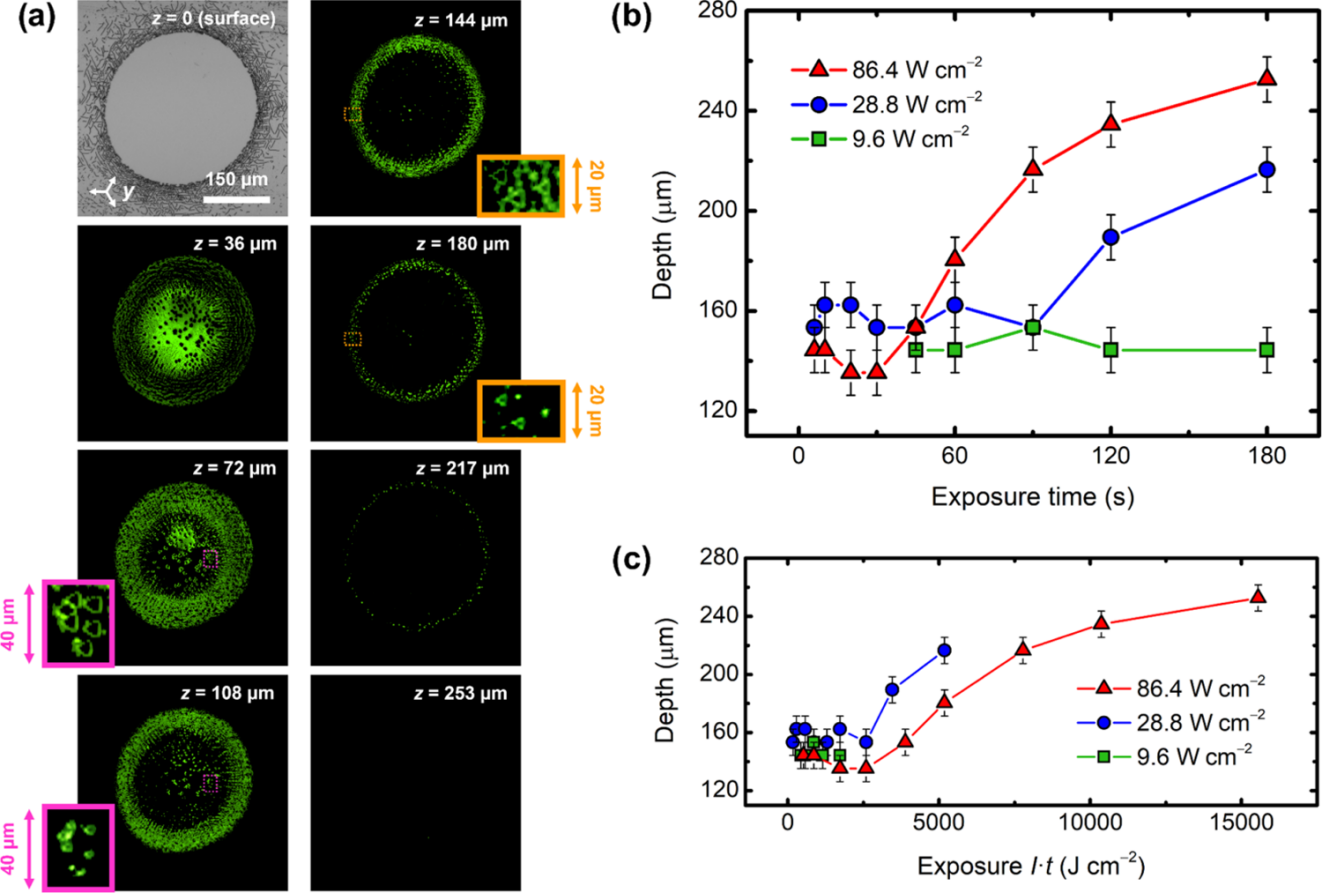
(a) Representative measurements of one of the inverted spots of Figure 3 (*I* = 86.4 W cm^–2^, *t* = 120 s) at different depths using a Čerenkov SHG microscope. The image for *z* = 0 corresponds to etching at the surface. Green areas indicate the Čerenkov SHG signal coming from domain walls. The scale bar is equal for all of the images at different depths, except for the insets. The pink and orange insets are magnified areas from two different regions. The graphs show the maximum depth reached by the domains as a function of (b) exposure time and (c) exposure. The solid lines have been plotted as a guide to the eye.

The results of the Čerenkov SHG scans are shown in Figure 4. A 3D stack is also provided in Figure S10 in the Supporting Information. First of all, Figure 4a reveals a rather complex domain structure in the bulk of the Fe:LN crystal. On the one hand, the “tentacles” around the inner circle are shallow, so we could not reliably measure the depth in our setup. The same applies to the nanoscale maze structures observed at low exposures (see Figure 2a). On the other hand, the inner circle may be subdivided into two parts: the central region and the outer ring. The central region comprises a large density of “spike” domains, even though it is uniformly inverted at the surface. The insets show the in-plane triangular shape of some of these domains, all of them oriented with the corners in the +*y* directions as usual, consistent with the etching results. The size of these triangles diminishes with depth and can typically go as deep as ∼150 μm. In contrast, the outer ring is only observed when the exposure is long enough and it can go deeper than 150 μm (see Figure 4a). This ring is also an ensemble of densely packed spike domains, having in-plane triangular shape. The deeper into the crystal volume, the thinner the ring gets until it vanishes. In these illumination conditions, this ring has reached a maximum depth of 253 ± 9 μm for the highest intensity and longest exposure time, as shown in Figure 4b. We believe that this ring of enhanced depth could be related to the accumulation of PV charge at the edge of a Gaussian beam, revealed by numerical simulations under ideal open-circuit conditions in ref (69) due to lateral currents. However, it should be noted that a detailed numerical analysis of the PV charge transport equations is still lacking in the literature to confirm the validity of this mechanism in screening conditions.

In Figure 4b,c, the maximum depth of the domains is plotted as a function of exposure time and exposure, respectively. Overall, the general trend of the axial growth along the *z-*direction is somewhat similar to the surface lateral growth studied in Figure 3. However, an interesting property is that the maximum depth is not solely governed by exposure alone (*E* = *I*·*t*), unlike the diameters of Figure 3. In other words, intensity and time do not seem to play a fully interchangeable role when it comes to the domain depth, since not all experimental data are superimposed in Figure 4c. This result suggests that, aside from the bulk PV effect in the one-center regime, there are additional contributions involved in the axial growth of the light-inverted domains along the polar direction *z*. There are two possible contributions: a second PV center and the pyroelectric effect. First of all, it is known that above intensities of ∼1000 W cm^–2^, the saturation PV field becomes intensity-dependent due to Nb_Li_ antisites, which act as a second PV center apart from Fe impurities.^46,66,70^ As a result, the time evolution is no longer determined by exposure alone. In our experiments, this threshold intensity is not reached at the −*c* face, but it is indeed surpassed at the incident +*c* face up to a certain depth into the crystal volume, due to a smaller spot size caused by the beam divergence and a lower absorbance. Also, in this intensity regime, the Fe:LN crystal could be locally heated up due to absorption, leading to pyroelectric effect. Nevertheless, further work is necessary to clarify this matter.

### Influence of the Laser Spot Size

**Figure 5:**
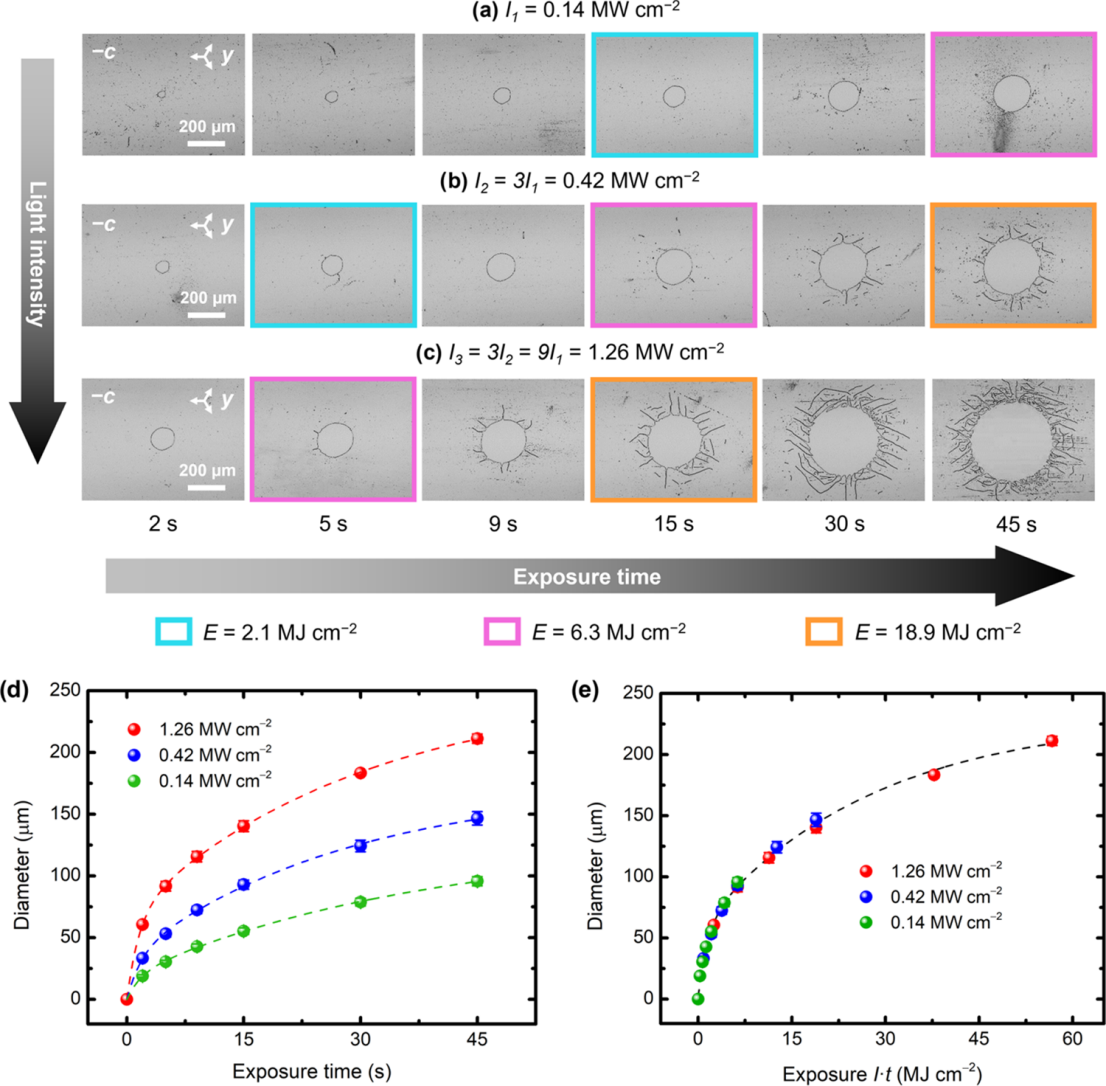
(a–c) Bright-field images of 18 domain inversion experiments using different light intensities and exposure times (after etching). In this case, the waist of the focused laser beam was located at the −*c* face, as illustrated in Figure S11a. Each row corresponds to a different light intensity (*I*) and each column to a different exposure time (*t*). The images with equally colored frames correspond to results with identical exposures (*E* = *I*·*t*). In the bottom graphs, the diameter of the inner circular domains is plotted as a function of (d) exposure time and (e) exposure. The dashed lines are fits to a function *f*(*x*) = *A*_1_·(1 – *e*^–*x*/*x*_1_^) + *A*_2_·(1 – *e*^–*x*/*x*_2_^), where *x* is either exposure time in (d) or exposure in (e). The fits are only included as a visual guide.

The impact of tighter light focusing conditions was also explored. For that purpose, the waist of the laser beam was focused on the −*c* face of the crystal, as shown in Figure S11a. Since the effective numerical aperture in our setup is NA_eff_ = 0.15, the 1/*e*^2^ diameter of the diffraction-limited light spot is estimated as *d* = 2λ/πNA_eff_ = 2.3 μm. The Rayleigh length can also be estimated as *z*_R_ = *n*_o_λ/πNA_eff_^2^ = 17 μm, where *n*_o_ = 2.32 is the ordinary refractive index of LN at 532 nm. Due to beam divergence, there is a strong variation of the light distribution along the *c*-axis, and the spot diameter at the +*c* face is 130 μm. In this configuration, domain inversion was only observed at the −*c* face (the bottom face), consistent with the behavior so far. The etching results are shown in Figure 5 for different light intensities and exposure times.

In this case, the morphology of the optically inverted domains differs from that of Figure 2 with a larger weakly focused laser spot. For low exposures, quasi-circular domains are formed, with growing diameters over time (see Figure 5a,b). The domain wall is not perfectly rounded but full of small steps generated during lateral growth (see Figure S11b). From a practical point of view, the ability to create clean quasi-circular domains, unencumbered by adjacent haloes, provides valuable added flexibility. In contrast, for long exposures, a halo of “tentacles” begins to appear at the border of the circle, somewhat similar to those of Figure 2. The number and length of tentacles increase with exposure time. Therefore, light focusing conditions provide another route to control the shape of inverted domains.

Another significant observation is that, even in this regime of high intensities (∼MW cm^–2^) several orders of magnitude higher than in Figure 3, the time evolution of the domain diameters is still governed by exposure (*E* = *I*·*t*), both qualitatively and quantitatively, as evidenced in Figure 5d,e. Thus, intensity and exposure time play an equivalent role, a common feature of the bulk PV effect. Nonetheless, it should be clarified that the exposure required for a given domain size may change when comparing different illumination configurations, e.g., Figure 3 versus Figure 5. This is attributed to the size and divergence of the focused laser beam along the thickness of the crystal, which leads to different light gradients along the *c*-axis depending on the focusing conditions. It is also worth noting that in Figure 5d,e, unlike Figure 3d,e, no threshold for the formation of quasi-circular domains is visible. This is because at these high intensities, this threshold exposure is reached in a very short time scale.

For further characterization, we also made additional Čerenkov SHG measurements, included in the Supporting Information in Figure S12. In this configuration, a remarkable maximum depth of 289 ± 9 μm was measured.

## Role of the Surrounding Medium

**Figure 6:**
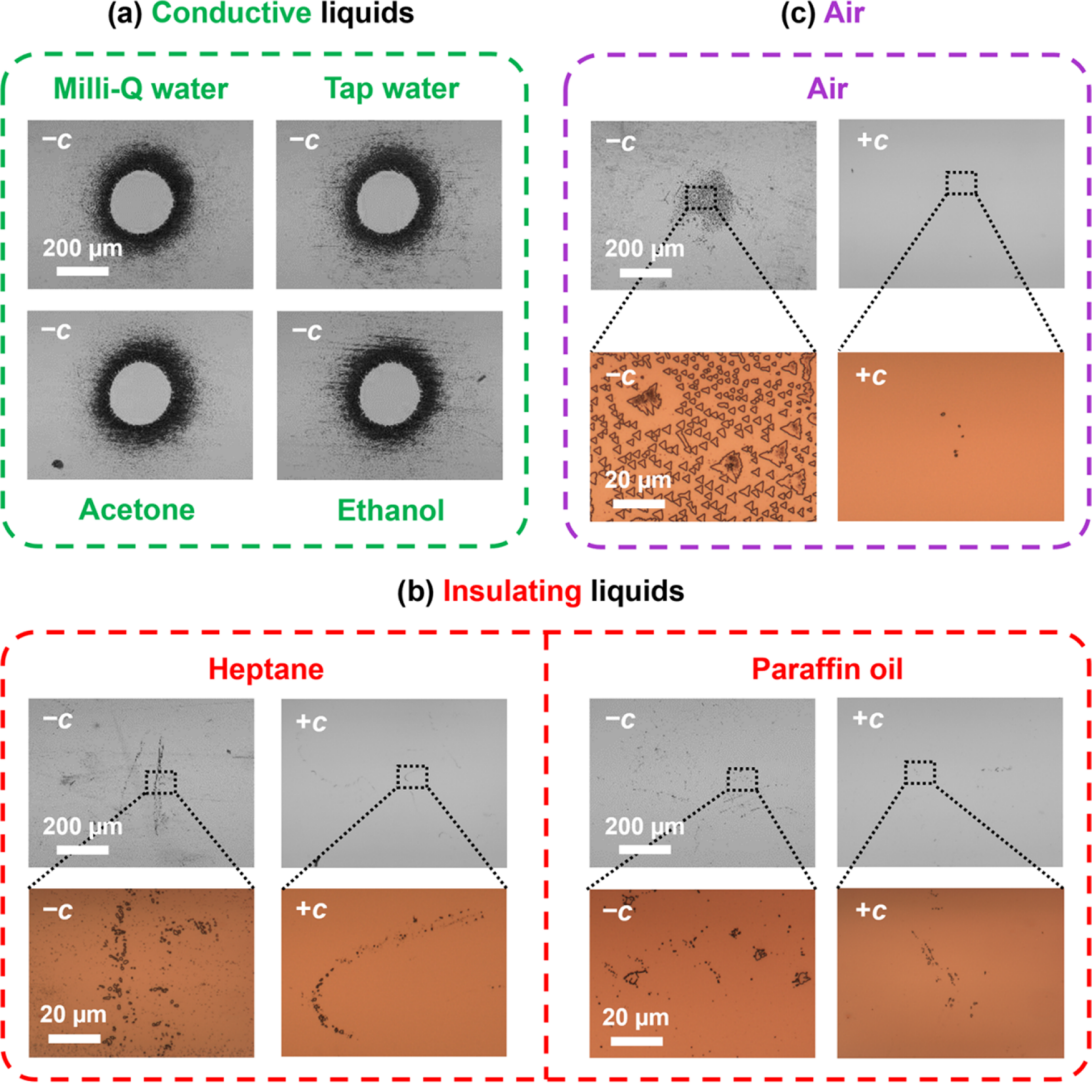
Bright-field images after etching of several experiments in different surrounding media: (a) conductive liquids, (b) insulating liquids, and (c) air. In all cases, the exposure time is 3 min, the incident optical power is 81 mW and the light spot is at the center of the images (with a diameter of *d* = 280 μm at the −*c* face, as explained in the experimental section, and *I* ≈ 15 W cm^–2^). The brown-colored images correspond to magnified areas of the wide-field grayscale images. The scale bar of 200 μm is equal for all of the grayscale images, whereas the scale bar of 20 μm applies to all of the brown-colored magnified insets.

So far, all of the results presented in the paper were obtained with Milli-Q water surrounding the Fe:LN crystal during the irradiation process. In this section, we compare different surrounding media (see Figure 6). First of all, equivalent results were obtained when using Milli-Q water, tap water, acetone (Uvasol, purity ≥99.9%), or ethanol (PanReac AppliChem, purity ≥99.9%), as shown in Figure 6a and Video S3. More specifically, there is a uniform inverted circle in the middle with a “halo” of closely packed domains at the border, in agreement with the behavior described in previous sections. These four liquids have in common that they are electrically conductive (with conductivities ≳10^–6^ S m^–1^) and, therefore, fast electrostatic screening. Conversely, a radically different behavior was observed when using highly insulating liquids with low electrical conductivity, such as *n*-heptane (PanReac AppliChem, purity ≥99.0%) or liquid paraffin oil (PanReac AppliChem, pure), as shown in Figure 6b. In this case, only a residual amount of new micro/nanodomains appeared in the irradiated region, having varied irregular shapes and located at seemingly random locations. Moreover, marginal domain inversion was also noticed at the +*c* face. Likewise, air (with a relative humidity of 28 ± 1%) shows a similar behavior, but a larger number of triangular microdomains is induced at the light spot (see Figure 6c).

Hence, these experiments reveal the critical role of the surrounding medium in the inversion process. We attribute this behavior to the electrical conductivity of the medium, which controls the external screening of the light-induced electric fields in the Fe:LN ferroelectric crystal. In fact, the residual amount of domain inversion observed in low-conductivity media could be due to dielectric breakdowns, which would make the medium conductive for a short amount of time until the evanescent electric field is rapidly screened.

## All-Optical Domain Inversion in Undoped Congruent LiNbO_3_

Finally, we explored the possibility of performing all-optical domain inversion in undoped congruent LN crystals. First, we used the same illumination conditions as in Figure 1, with a spot diameter of about 280 μm at the bottom face (the −*c* face). Using the maximum output power of the laser, a maximum intensity of ∼1.5 kW cm^–2^ could be reached with this spot size. However, no domain inversion whatsoever was observed for exposure times up to 45 s, i.e., exposures up to 68 kJ cm^–2^. Note that this is a relevant difference compared to Fe:LN crystals, where much lower intensities can easily induce domain inversion.

**Figure 7:**
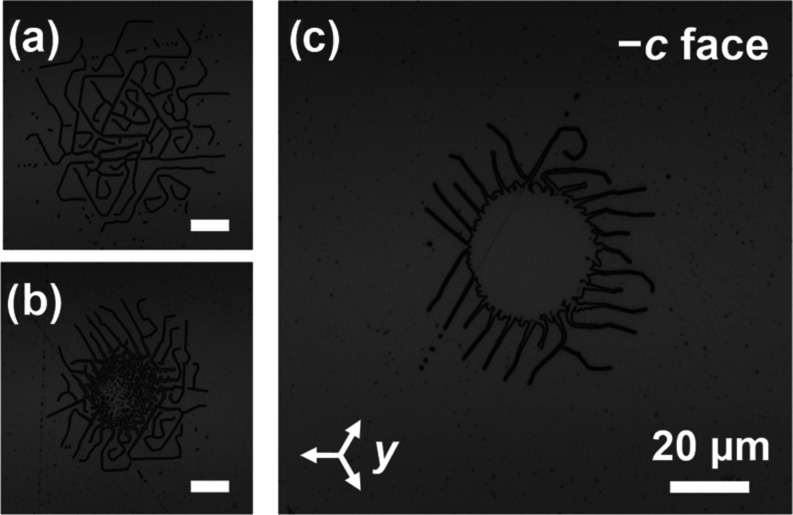
Bright-field images after etching of two domain inversion experiments in undoped congruent LN. Each experiment corresponds to a different laser spot size but equal incident optical power: (a) *d* = 94 μm, *I* = 5.9 kW cm^–2^, (b) *d* = 60 μm, *I* = 14.5 kW cm^–2^, (c) *d* = 36 μm, *I* = 40.3 kW cm^–2^. In all cases, the exposure time is 45 s. The scale bar represents 20 μm.

Then, due to the limitation of the laser output power, we progressively decreased the laser spot size in order to further increase the light intensity. By doing so, we were indeed able to locally invert domains, as illustrated in Figure 7. For example, for an intensity of 5.9 kW cm^–2^, we observed a self-assembled maze structure (see Figure 7a), rather similar to the results with Fe:LN in the low-exposure regime (see Figure 2a). In this structure, the *y* directions are somewhat favored. As the intensity increases, the branches of the maze become thicker at the center of the irradiated area and begin to coalesce (see Figure 7b). Eventually, a uniformly inverted quasi-circular domain is generated at the center, with a halo of tentacles preferentially pointing in the −*y* directions (see Figure 7c). In general, these structures are quite similar to Fe:LN in Figures 3 and 5, and they are generated only at the −*c* face. Thus, the general trend is similar to Fe:LN. Therefore, this technique is not just restricted to Fe:LN, but it can be generalized to undoped congruent LN as well. A more detailed experimental analysis of undoped congruent LN will be tackled in future work, including the depth and the role of exposure time.

## Discussion on the Physical Mechanism of Domain Inversion

In our experiments, there could be two main sources of light-induced electric fields: the bulk PV effect and the pyroelectric effect (due to absorption). Experimental evidence suggests that the bulk PV effect is the main driving agent because:

(1) Domain inversion can be achieved even at low light intensities, when the crystal heating is expected to be negligible. So far, we have observed domain inversion with intensities as low as ∼200 mW cm^–2^ (incident power of ∼1 mW) at the expense of increasing the exposure time accordingly (*t* = 5 min or longer). This is attributed to the continuous supply of PV current under light excitation, unlike the pyroelectric effect, which is not relevant at such intensities.
(2) Domains can also be inverted in undoped congruent LN, where the absorption of a CW visible laser at the intensities of this work is negligible for heating purposes. In nominally undoped LN, trace impurities such as Fe or Cu (on the order of ∼ ppm) and intrinsic defects (namely, Nb_Li_ antisites) are known to be responsible for the bulk PV effect.^66,72^ Comparatively, PV currents are much lower than in Fe:LN. On the other hand, the contribution of the antisites leads to a saturation PV field that grows with light intensity, unlike Fe:LN where the saturation field remains constant in the one-center regime (i.e., low intensities). At high light intensities, open-circuit PV fields in undoped congruent LN become close to those of Fe:LN, reaching values up to ∼200 kV cm^–1^ at ∼10^6^–10^7^ W cm^–2^.^46^ Thus, the inherently weaker PV effect in undoped LN could account for the higher intensities needed to invert domains compared to Fe:LN.
(3) The lateral growth of the inverted domains in Fe:LN is governed by exposure (*E* = *I*·*t*) in each illumination configuration, as shown in Figures 3 and 5.

However, PV electric fields in the usual open-circuit conditions (i.e., when the crystal is surrounded by an insulating environment) are parallel to the spontaneous polarization. In general, the time evolution of PV charge transport is governed by the well-known photorefractive rate equations.^71^ In the one-center model for Fe:LN, iron impurities are the only PV center responsible for the bulk PV effect. Namely, electrons undergo directional optical transitions from Fe^2+^ donors to the conduction band due to the noncentrosymmetric crystal lattice, giving rise to a PV current along the ferroelectric *c*-axis. Subsequently, electrons recombine with Fe^3+^ acceptors. In this widespread model, the light-induced PV current may be written as follows:
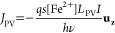
1where *q* is the electron charge in absolute value, *s* is the photoionization cross section of Fe^2+^, [Fe^2+^] is the concentration of Fe^2+^ donors, *L*_PV_ is the PV transport length, *I* is the light intensity, *h* is Planck’s constant, ν is the light frequency, and **u**_**z**_ is a unit vector along the *c*-axis of the Fe:LN crystal. In ideal open-circuit conditions, the surrounding medium is considered to be a perfect electrical insulator. Thus, PV charge is accumulated at the surface of *z*-cut crystals until saturation is reached. In particular, positive charge is induced at the −*c* face due to an accumulation of Fe^3+^ acceptors, while negative charge is induced at the +*c* face due to an accumulation of photoexcited carriers and Fe^2+^ donors. The redistributed charge generates a growing electric field that opposes the PV current, inducing the saturation of the process. Assuming homogeneous light intensity and neglecting diffusion, the time evolution of the PV electric field inside the crystal is given by the usual monoexponential growth:^69^
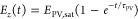
2where *E*_PV,sat_ = γ*L*_PV_[Fe^3+^]/μ is the saturation electric field, τ_PV_ = ε_33_ε_0_γ[Fe^3+^]*h*ν/μ*qsI*[Fe^2+^] is the PV response time, γ is the recombination constant, μ is the electron mobility, ε_33_ is the corresponding relative permittivity of Fe:LN, ε_0_ is the vacuum permittivity, and [Fe^3+^] is the concentration of Fe^3+^ acceptors. Notably, in open-circuit conditions, *E*_*z*_ is always parallel to the spontaneous polarization.

**Figure 8:**
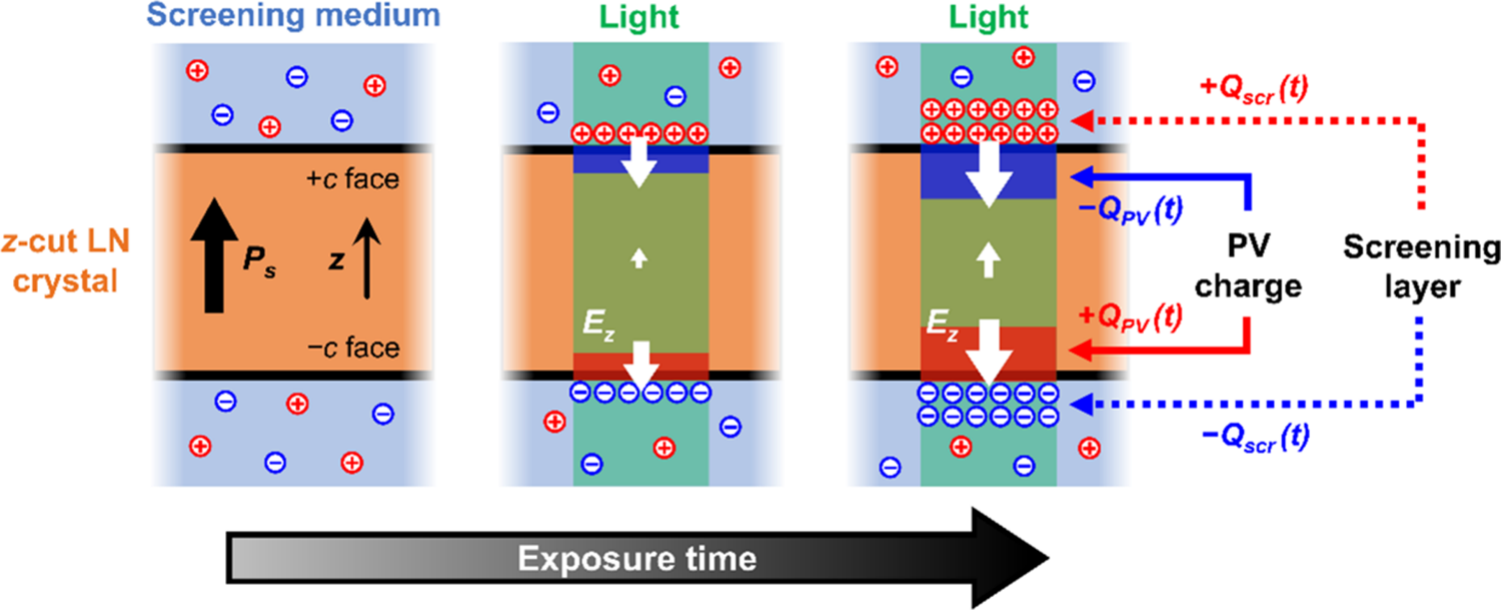
Schematic diagram of the proposed mechanism for light-induced ferroelectric domain inversion driven by the bulk PV effect. The illustration shows the PV charge transport under external electrostatic screening. The sketch includes the spatial distribution of PV charge in the LN crystal (*Q*_PV_), the screening charge of the surrounding medium (*Q*_scr_), and the net electric field (*E*_*z*_).

However, in this work on domain inversion, the Fe:LN crystal is surrounded by an electrically conductive medium (mainly Milli-Q water, with a nominal resistivity of 18.2 MΩ·cm at room temperature). Therefore, external electrostatic screening cannot be neglected. In fact, based on the results of Figure 6, it is expected that the crystal–liquid screening interface plays a critical role in the inversion process. Nevertheless, PV fields under strong screening remain poorly understood. In Figure 8 we propose a novel physical mechanism for all-optical domain inversion, based on the interplay between the bulk PV effect and external screening. Here, the light intensity is assumed to be homogeneous for simplicity. Upon light excitation, a PV current is generated, and PV charge starts to build up at the +*c* and the −*c* faces. In response, a screening current is induced in the surrounding medium to compensate the PV charge. As a result, screening charges of opposite sign will be adsorbed at the surface of the crystal. In the case of Milli-Q water, an excess of OH^–^ negative ions and H^+^ positive ions is expected at the −*c* face and the +*c* face, respectively, forming a Debye screening layer (see Figure 8). Since the stored PV charge is continuously screened, the charge separation process does not saturate as it occurs in open-circuit conditions. Consequently, during illumination, PV charge will be continuously pumped toward the crystal surface at a rate given by the PV current.

Importantly, PV charge is stored in bulk iron traps. Therefore, iron donors will turn into Fe^3+^ acceptors near the −*c* face, while iron acceptors will turn into Fe^2+^ near the +*c* face. Since iron traps are finite in number, the region where the PV charge is stored will progressively grow over time from the crystal surface toward the bulk, as depicted in Figure 8. Since PV charge is not strictly located at the surface, the PV field cannot be fully screened inside the crystal by the external medium. Thus, there will be a net electric field *E*_*z*_, which is the sum of the PV contribution and the screening contribution. A novel outcome of this synergy is that the net electric field within the space charge region will be antiparallel to the spontaneous polarization, as dictated by the external screening charges (i.e., *E*_*z*_ < 0, opposite to the usual direction in open-circuit conditions). Moreover, in perfect screening conditions, the electric field outside the crystal should vanish, and so, the surface of the crystal should be equipotential. This boundary condition implies that there is no potential difference between the +*c* and the −*c* face: Δ*V* = ∫_–*c*_^+*c*^*E*_*z*_·d*z* = 0. To fulfill this condition, there must be a residual electric field *E*_*z*_ > 0 in the volume of the crystal as depicted in Figure 8. Neglecting this marginal component in the volume, the electric field at the surface under ideal screening conditions can be estimated using an infinite parallel-plate capacitor approximation:
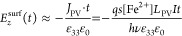
3where *J*_PV_ is the PV current density given by eq 1. Note that, in these conditions, *E*_*z*_^surf^ is antiparallel to the spontaneous polarization as required to invert domains, and it can reach values higher than the saturation field in open-circuit conditions (*E*_PV,sat_). Therefore, we propose that *E*_*z*_^surf^ grows with exposure (*I*·*t*) due to the accumulation of PV and screening charge, until it exceeds the coercive threshold needed for nucleation and growth of inverted ferroelectric domains. In summary, our analysis leads us to consider the singular synergy between the bulk PV current and the external screening by the conductive surrounding medium as the main physical mechanism for all-optical domain inversion by visible light.

It is worth mentioning that certain features have been omitted from this model for simplicity. First of all, in our experiments the light intensity is not homogeneous due to the Gaussian beam profile, the beam divergence, and crystal absorption. Furthermore, at the −*c* face, a gradual depletion of Fe^2+^ donors is expected. Hence, the PV current and the photoconductivity will dwindle over time. In contrast, an excess of Fe^2+^ donors and photoexcited carriers will accumulate at the +*c* face. In this framework, stronger carrier diffusion would be expected at the +*c* face. Thus, the asymmetric distribution of Fe impurities and diffusion could play a relevant role in the charge transport, which is not considered in Figure 8. Relatedly, diffusion has already been invoked in the literature to account for the asymmetric behavior of the +*c* and −*c* faces^31,32^ commonly observed in light-assisted poling of LN.^27^ On the other hand, when the coercive threshold is reached and the onset of domain inversion is triggered, polarization charges will switch, and the direction of the PV current will be reversed. These contributions could also have an influence on domain growth kinetics. Finally, at this stage, a residual influence of the pyroelectric effect cannot be ruled out. Further work is necessary to unravel the role of these additional factors in our physical mechanism, which is beyond the scope of this first report on the subject.

## Conclusions

Overall, this paper encompasses a set of solid proofs that demonstrate for the first time all-optical ferroelectric domain inversion of Fe:LN and undoped LN crystals with visible light. According to our results, the successful realization of our method strongly relies on the presence of a conductive surrounding medium during irradiation. Based on this finding, the underlying physical mechanism is attributed to an anomalous interplay between the bulk PV effect and interfacial electrostatic screening from the surrounding medium. A simple qualitative model can explain the optical induction of PV fields antiparallel to the spontaneous polarization. Such electric fields grow with exposure due to the accumulation of screening charges until the coercive field is surpassed, leading to ferroelectric domain inversion. Furthermore, the experimental results show that the size and morphology of the inverted domains can be flexibly controlled by the light intensity, exposure time, or focusing conditions, all the way from self-assembled maze structures to quasi-circular domains. Domains with a maximum depth of 289 ± 9 μm have been measured, deeper than any other IR/UV all-optical technique to date without crystal damage. Nonetheless, these depths are still lower than those attainable by electrical poling, capable of producing domain depths in the millimeter range.

The method is simple and easy to implement, not requiring any lithography-patterned electrodes or external voltage supplies. Moreover, since the bulk PV effect of LN can be excited in the full visible spectrum, there is a broadband range of suitable light sources compatible with the method. In fact, incoherent light sources with moderate optical powers could also be employed. Likewise, spatial light modulators could be readily included in the setup to tailor complex light patterns, aiming at the parallel fabrication of arbitrary ferroelectric domain structures.

These domain structures are of practical interest for a number of fields, such as photonics or domain-wall nanoelectronics/optoelectronics. Also, optically patterned domains could be used as templates for mask-free lithography of Fe:LN and LN surfaces via selective wet etching. For instance, LN metasurfaces may be envisioned.^6^ On the other hand, although iron-doped LN is not the preferred platform for nonlinear photonics, this material stands out for its prominent bulk PV effect. This phenomenon makes Fe:LN an ideal substrate for the so-called “optoelectronic tweezers”,^56^ a light-assisted technique aimed at the versatile manipulation, trapping, and patterning of micro/nano-objects. In this context, PV optoelectronic tweezers could greatly benefit from a completely new degree of freedom not explored so far: multidomain Fe:LN substrates. This feature could enhance the already existing applications or pave the way toward new functionalities of ferroelectric PV platforms.

## Supporting Information Available

Additional experimental details and results, schematic diagram of the full optical setup employed for light-induced domain inversion of LN crystals, high-resolution SEM images, in-depth analysis of the orientation of the inverted domains, details about Čerenkov SHG measurements, and additional characterization (PDF)
Real-time video of the irradiation of a Fe:LN crystal (Video S1) (MP4)
Real-time video of the irradiation of a Fe:LN crystal for several light intensities (Video S2) (MP4)
Real-time video of the irradiation of a Fe:LN crystal surrounded by several liquid media with high electrical conductivity (Video S3) (MP4)
