# Supplementary material for: All-Optical Domain Inversion in LiNbO3 Crystals by Visible Continuous-Wave Laser Irradiation
Source: ACS Photonics. 2024 Jun 21;11(7):2624–36. doi: 10.1021/acsphotonics.4c00336 (PMC11258989; doi:10.1021/acsphotonics.4c00336)
Supplement: Supplementary file 1 — ph4c00336_si_001.pdf [file ph4c00336_si_001.pdf]

Supporting Information

All-Optical Domain Inversion in $LiNbO_3$ Crystals by Visible Continuous-Wave Laser Irradiation

Carlos Sebastián-Vicente,[1,2] Jörg Imbrock,[3] Simon Laubrock,[3] Olga Caballero-Calero,[4] Angel García-Cabañes,[1,2] and Mercedes Carrascosa[1,2,]*

[1]Departamento de Física de Materiales, Universidad Autónoma de Madrid, 28049 Madrid, Spain

[2]Instituto Nicolás Cabrera, Universidad Autónoma de Madrid, 28049 Madrid, Spain

[3]Institute of Applied Physics, University of Münster, Corrensstr. 2, 48149 Münster, Germany

[4]Instituto de Micro y Nanotecnología, IMN-CNM, CSIC (CEI UAM+CSIC) Isaac Newton, 8, Tres Cantos, E-28760 Madrid, Spain

*E-mail of corresponding author: m.carrascosa@uam.es

Number of pages: 12
Number of figures: 12
Number of tables: 0

A. Full Optical Setup and Further Experimental Details

A schematic diagram of the experimental setup employed for all-optical domain inversion is shown in Figure S1. Note that the LN crystal is actually immersed in a cuvette as shown in Figure 1, typically filled with Milli-Q water unless otherwise specified.

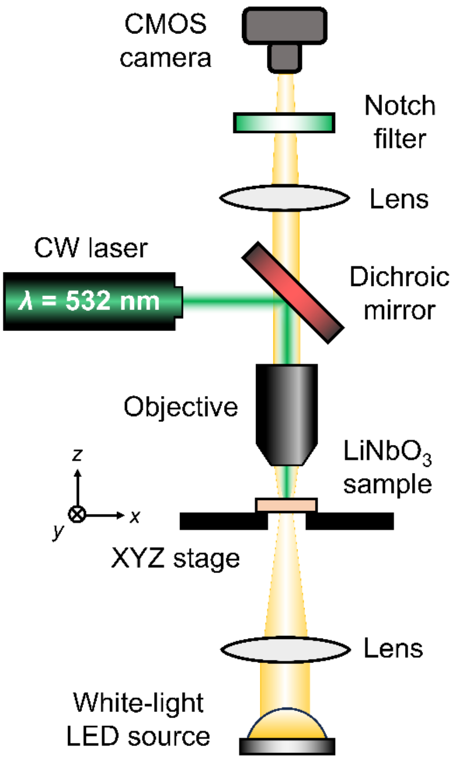

Figure S1. Complete experimental setup for all-optical domain inversion.

Regarding light intensities, they are always specified at the bottom face of the crystal, i.e. the −*c* face where domain inversion takes place in our experimental conditions. This means that losses due to Fresnel reflections and absorption along the crystal thickness should be taken into account. Intensities are calculated with the following expression:

$$I = \frac{P \cdot (1 - R_{aw})(1 - R_{wc}) e^{-\alpha_{abs} h}}{\pi (2\sigma)^2} \tag{S1}$$

where P is the incident optical power before losses (measured with a thermopile), R_{aw} is the Fresnel reflectance at the air-water interface, R_{wc} is the Fresnel reflectance at the water-crystal interface, α_{abs} is the absorption coefficient of the LN crystal at 532 nm, h is the crystal thickness and 2σ is the $1/e^2$ radius of the Gaussian beam. Secondary Fresnel reflections are neglected. It is worth noting that the peak intensity of the Gaussian beam would be $2I$. The coefficients R_{aw} and R_{wc} can be computed using the Fresnel formula at normal incidence: $R = [(n_1 - n_2)/(n_1 + n_2)]^2$, where n_1 and n_2 are the refractive indices of the media forming the interface. This expression yields $R_{aw} = 0.02$ and $R_{wc} = 0.07$. On the other hand, the absorption correction factor in our

crystals is $e^{-\alpha_{\text{abs}}h} = 0.13$. Thus, for instance, for an incident power of 50 mW and a typical laser spot size of $d = 4\sigma = 280$ µm, the intensity would be $I = 9.6$ W cm^{-2}.

B. Supporting Videos

Video S1. Real-time video of the irradiation of a Fe:LN crystal ($I = 28.8$ W cm^{-2}, $t = 180$ s). The speed of the video has been increased by a factor 12X.

Video S2. Real-time video of the irradiation of a Fe:LN crystal for several light intensities: a) $I = 9.6$ W cm^{-2}, b) $I = 28.8$ W cm^{-2}, and c) $I = 86.4$ W cm^{-2}. In all cases the exposure time was $t = 90$ s. The speed of the video has been increased by a factor 6X.

Video S3. Real-time video of the irradiation of a Fe:LN crystal surrounded by several liquid media with high electrical conductivity: a) Milli-Q water, b) tap water, c) acetone, and d) ethanol. In all cases the exposure time was $t = 180$ s and the incident optical power was 81 mW. The speed of the video has been increased by a factor 12X.

In Videos S1-S3 (transmission bright-field microscopy) the domain inversion process can be observed in real time at the non-incident face of the crystal (i.e. the $-c$ face). Snapshots of Video S1 are given in Figure S2 as an example. Note, however, that the microscope image is focused at the upper incident face (i.e. the $+c$ face). As a result, the optical image is not sharp at the face where light-induced domain inversion occurs.

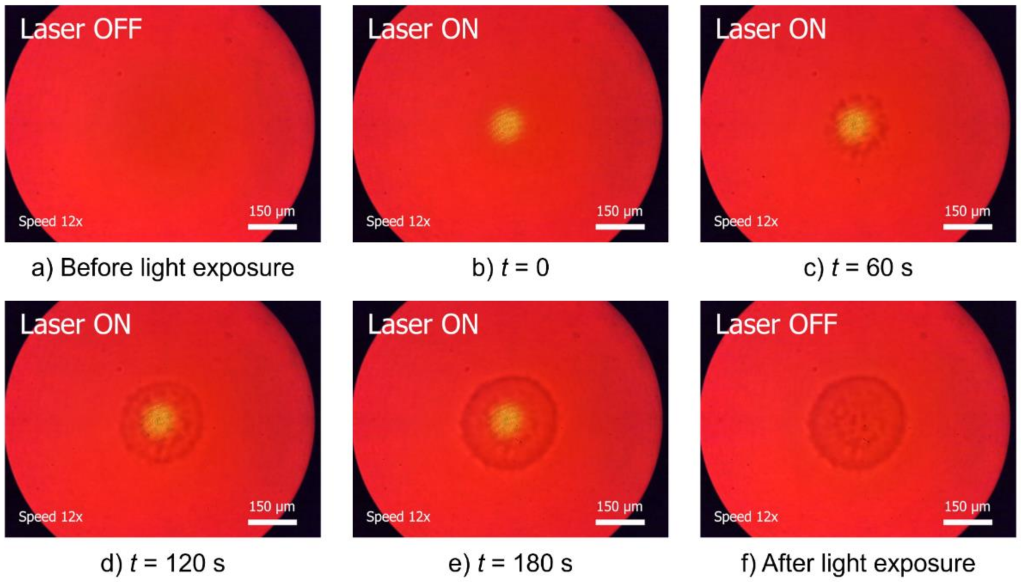

Figure S2. Sequence of snapshots corresponding to Video S1.

C. Selective Pyroelectric Trapping/Repulsion of Charged Microparticles

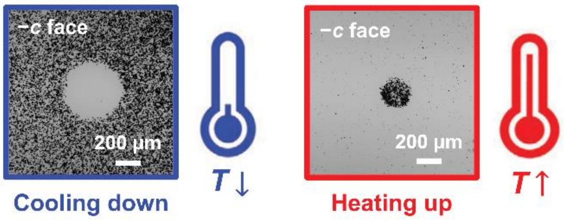

Figure S3. Trapping/repulsion of positively-charged toner microparticles induced by the pyroelectric effect at the light-inverted spot in Fe:LN (intensity $I = 86.4$ W cm^{-2}, exposure time $t = 1$ min). The inverted spot is the same as in Figure 1 (prior to etching). The left image shows the result upon crystal cooling ($\Delta T = -12\pm1$ °C), whereas the right image corresponds to crystal heating ($\Delta T = 12\pm1$ °C).

Aside from chemical etching, we also exploited the pyroelectric effect to decorate the light-induced ferroelectric domains using charged microparticles. Upon heating/cooling, the spontaneous polarization of LN decreases/increases, leading to net surface charges due to the imbalance between polarization and screening charges. Namely, negative/positive surface charges are induced at the +*c* face upon heating/cooling, whereas surface charges with opposite polarity are generated at the −*c* face.[13] This effect has been widely used for electrostatic micro/nanoparticle patterning in the literature.[19] To make sure that there was no influence whatsoever from PV space charges after the light excitation, these experiments were conducted weeks later, much longer than the dark lifetime of the PV fields in our Fe:LN crystals. We used toner microparticles extracted from the cartridge of a laser printer (Canon, C-EXV 29, black toner). The size of the toner particles is around ~1-10 µm and they carry positive net charge. Then, the toner particles were dispersed in an insulating liquid, namely *n*-heptane (PanReac AppliChem), using an ultrasonic bath. Before pyroelectric trapping, the particle suspension was cooled down in a fridge ($\Delta T = -12\pm1$ °C) or heated up on a heating plate ($\Delta T = 12\pm1$ °C). The temperature of the suspension was measured with an IR thermometer and a thermocouple, yielding the same result. Finally, the Fe:LN crystal with inverted domains (initially at room temperature, $T = 21\pm1$ °C) was immersed in the cold/hot suspension of particles for around 20 seconds. The results are shown in Figure S3. Upon cooling, the positively charged particles were repelled from the irradiated area and attracted everywhere else. However, the opposite behavior is observed upon heating. This finding strongly suggests that the −*c* face has been locally switched to +*c* by the laser. Also, the repulsion area upon cooling is larger than the trapping area upon heating, due to the additional electrostatic action of the non-inverted −*c* surface. Along with chemical etching, these results are solid evidence of all-optical domain inversion, revealing the light-induced reversal of the pyroelectric charge sign.

D. Scanning Electron Microscopy (SEM)

Due to the nanoscale feature sizes of some of the domain structures, high-resolution SEM images of the etched domains were acquired. Prior to coating the sample, the crystals were gently cleaned using lens-cleaning tissue soaked in distilled water mixed with soap (Fairy). The sample was finally rinsed with bare distilled water and the liquid residuals were blown away with compressed air. Then, a 10-nm-thick conductive coating of Chromium was deposited on the $-c$ face of the crystal by sputtering, to prevent charge accumulation during SEM inspection. Finally, a high-resolution SEM was employed (model Verios 460, from FEI).

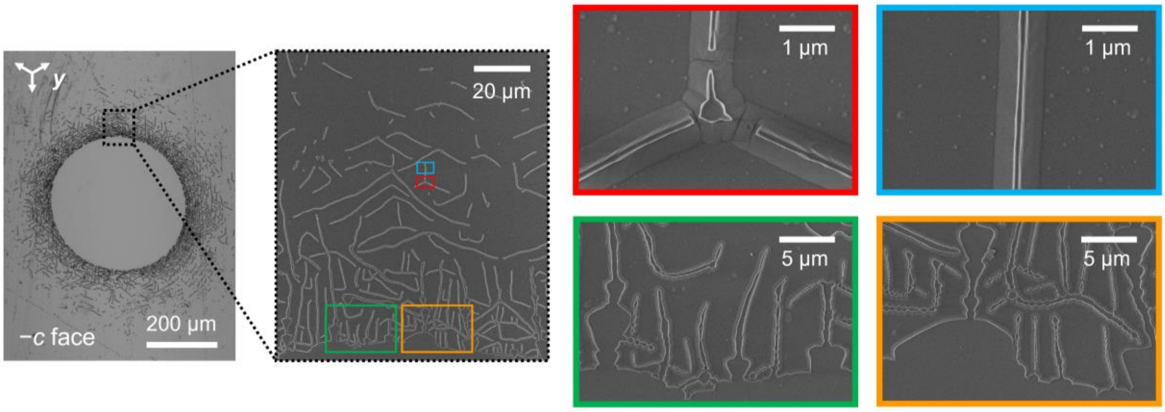

Figure S4. High-resolution details of the tentacle domains at the edge of a circular domain (intensity I = 86.4 W cm^{-2}, exposure time t = 2 min). The image on the left corresponds to the bright-field image after etching, while the others are SEM images at different magnifications.

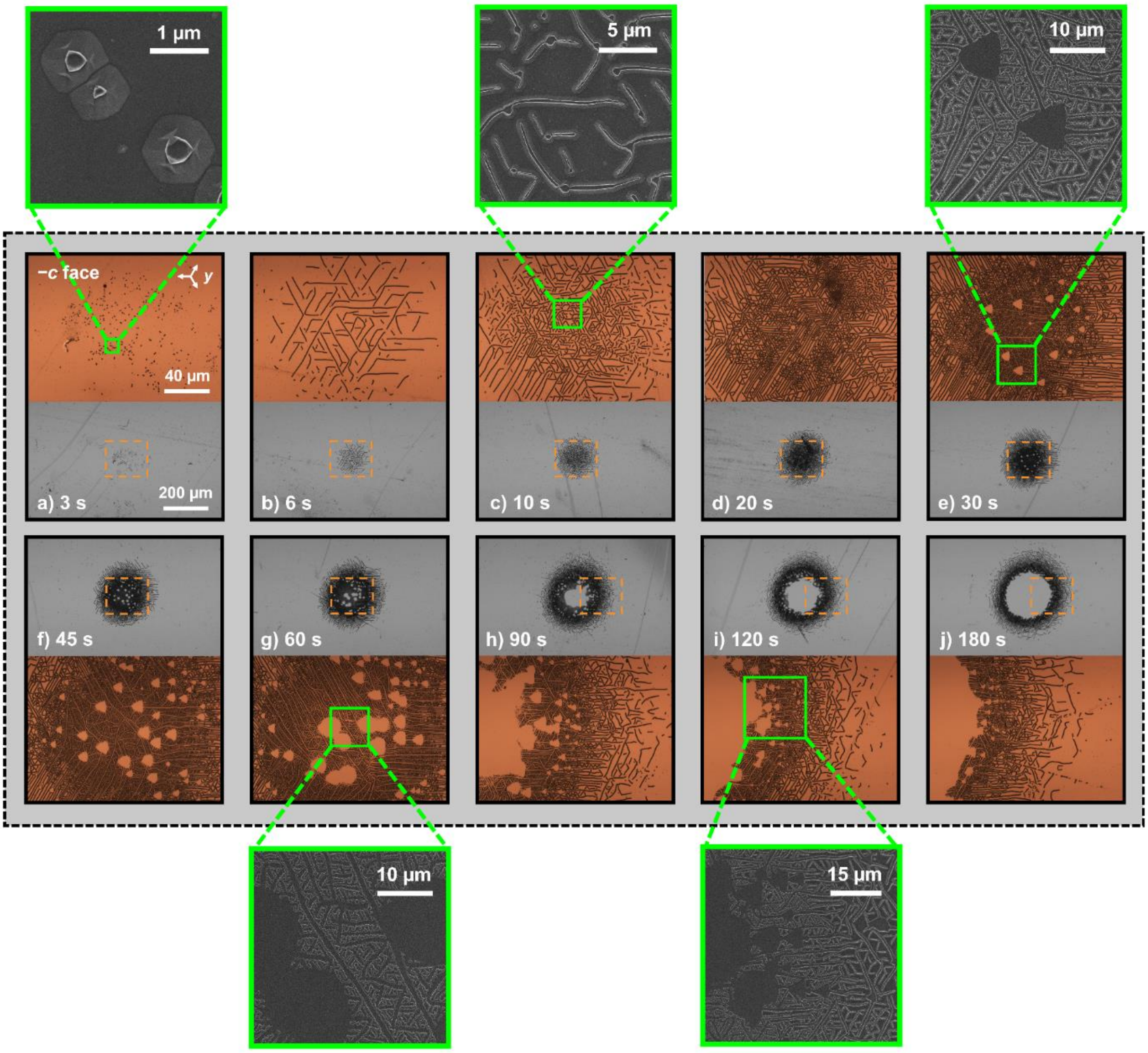

Figure S5. Micro-photographs (after etching) of light-induced inverted domains for different exposure times from a) 3 s up to h) 180 s. In all cases the light intensity is I = 9.6 W cm^{-2}. The brown-colored images correspond to magnified areas of the widefield grayscale images (indicated by a brown dashed rectangle). The green insets correspond to high-resolution SEM images.

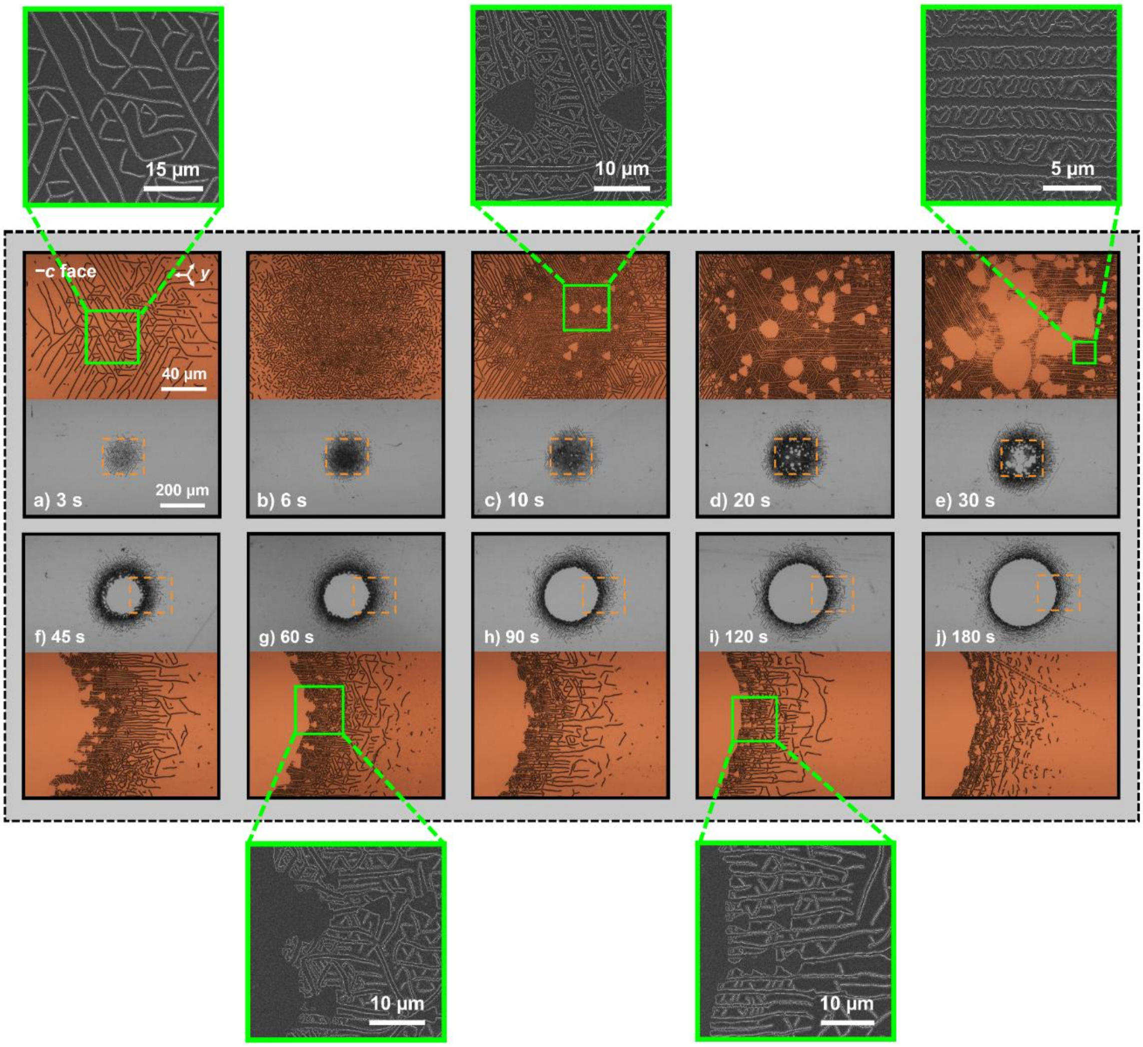

Figure S6. Micro-photographs (after etching) of light-induced inverted domains for different exposure times from a) 3 s up to h) 180 s. In all cases the light intensity is $I = 28.8$ W cm^{-2}. The brown-colored images correspond to magnified areas of the widefield grayscale images (indicated by a brown dashed rectangle). The green insets correspond to high-resolution SEM images.

E. Orientation of "Tentacle" Domains and Self-Assembled Maze Structures

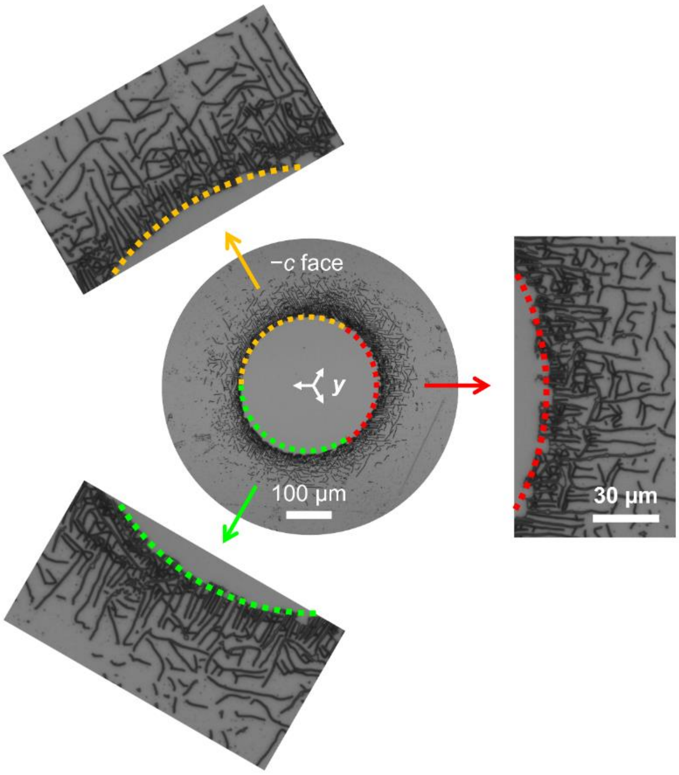

Figure S7. Illustration of the directionality of the "tentacle" domains located at the border of a circular domain (I = 86.4 W cm^{-2}, exposure time t = 1 min). Each sector of the halo with a given orientation is highlighted by a different color. There are three main orientations, coinciding with the −y crystallographic directions.

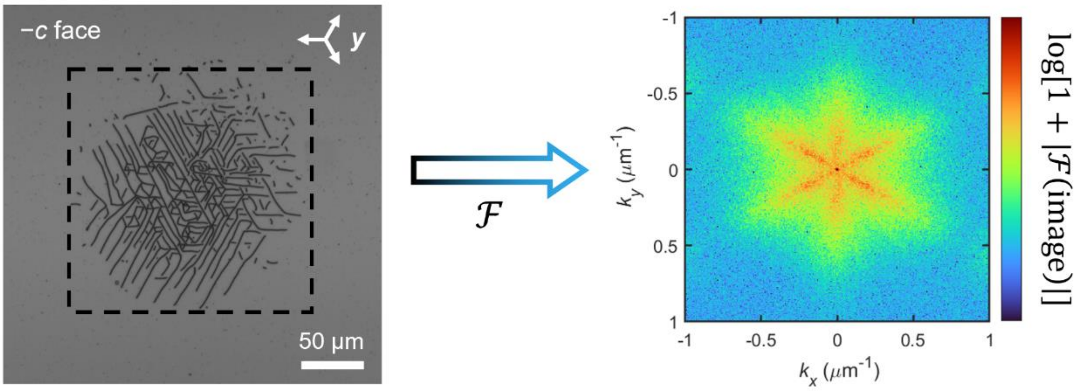

Figure S8. Example of a 2D Fourier transform (right) of a self-assembled maze structure of ferroelectric domains (left), corresponding to Figure 2a.

F. Čerenkov SHG Microscopy

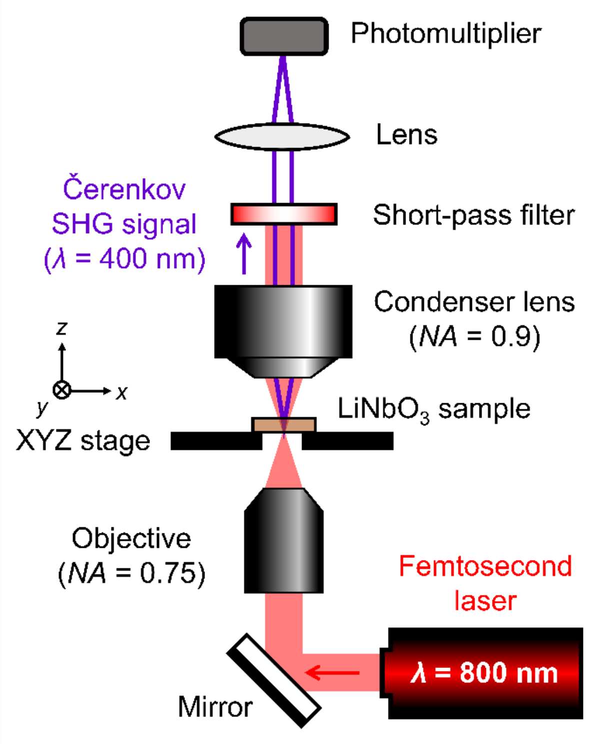

Figure S9. Sketch of the Čerenkov SHG microscope employed for 3D ferroelectric domain mapping.

The domain walls in Figure 4, S10 and S12 are detected using a Second Harmonic-Generation (SHG) laser scanning microscope consisting of a mode-locked Ti:sapphire laser (Vitesse, Coherent) and an inverted optical microscope (Nikon eclipse, Ti-U).[68] The setup is schematically depicted in Figure S9. The laser emits laser pulses at 800 nm with 80 MHz repetition rate, 90 fs pulse duration at an average power of 270 mW. The laser beam is expanded and fills the rear aperture of the microscope objective with a numerical aperture of 0.75. The laser power is attenuated by a combination of half-wave plate and polarizer to prevent all-optical domain inversion during the measurement.[41] The maximum power in the sample plane is below 100 mW (1.25 nJ pulse energy). The light is focused in the *z*-cut crystal mounted on a piezo stage (P-545, PI nano) with a *xyz*-travel range of 200 µm × 200 µm × 200 µm and a position resolution of 1 nm. The $1/e^2$ diameter of the diffraction-limited light spot is estimated as $d = 2\lambda/\pi NA$ = 680 nm. The crystal is moved through the laser focus in *xyz*-steps of typically 1 µm × 1 µm × 4 µm. Second harmonic light is collected by a condenser lens with a numerical aperture of NA = 0.9 and recorded by a photomultiplier (H6780, Hamamatsu) as a function of the focus position. The fundamental wave is effectively blocked by a short-pass filter. The piezo nano-stage is mounted on a *xy*-stage (M-545), which is moved by stepper motors with a travel

range of 25 mm × 25 mm and a minimum incremental motion of 1 µm. This allows for scanning of domains with a diameter larger than 200 µm (see Figure S10).

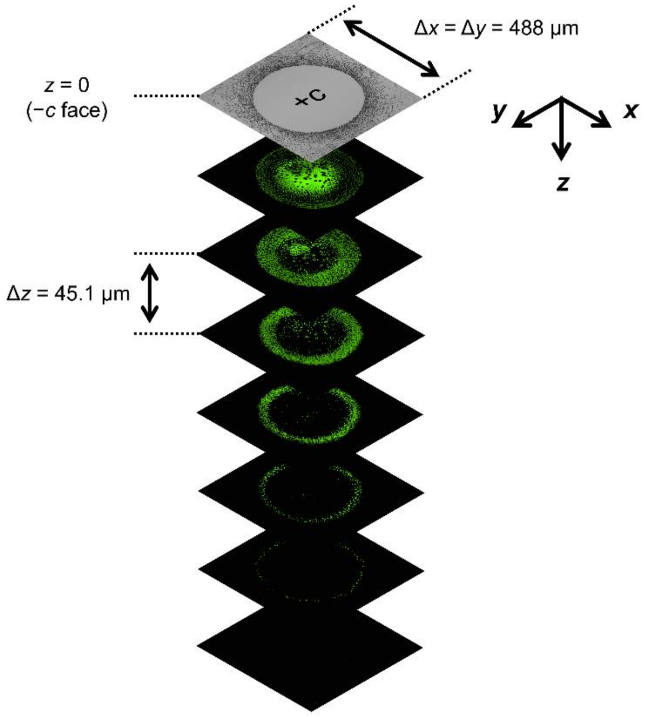

Figure S10. 3D stack of Čerenkov SHG scans of one of the inverted spots of Figure 3 (I = 86.4 W cm^{-2}, t = 120 s) at different depths using a Čerenkov SHG microscope. The image for z = 0 corresponds to etching at the surface. Green areas indicate the Čerenkov SHG signal coming from domain walls.

If the laser focus is located within the LN crystal in a volume without polarization changes, i.e. with a constant $\chi^{(2)}$ nonlinearity, no second harmonic wave is generated. The reason for this lies in the Gouy phase of the focused Gaussian laser pulse, which cancels out any second harmonic, as we are working in a dispersion regime with positive phase mismatch $\Delta k = k_{2\omega} - 2k_{\omega} > 0$, with the wave vector $k_{2\omega}$ and k_{ω} of the second harmonic and fundamental wave, respectively. However, if the laser beam is focused on an interface where the nonlinearity/polarization changes, a second harmonic wave is generated. This happens, for example, at the surface of the crystal, but also when the polarization in the crystal changes from $-P_s$ to $+P_s$ (or from $+P_s$ to $-P_s$), i.e. when the domain wall lies in the xy-plane. A second harmonic wave is then generated, which propagates mainly parallel to the z-axis. On the other hand, if the laser focus is located in a domain wall that lies in the z-plane, a second harmonic wave is generated that propagates at an angle θ determined by the longitudinal phase matching condition $\cos\theta = 2\,k_{\omega}/k_{2\omega}$, also known as Čerenkov-type phase matching.[67] To detect second harmonic waves at large Čerenkov angles, a condenser with a high NA is required.

G. Quasi-Circular Domains under Tighter Focusing Conditions

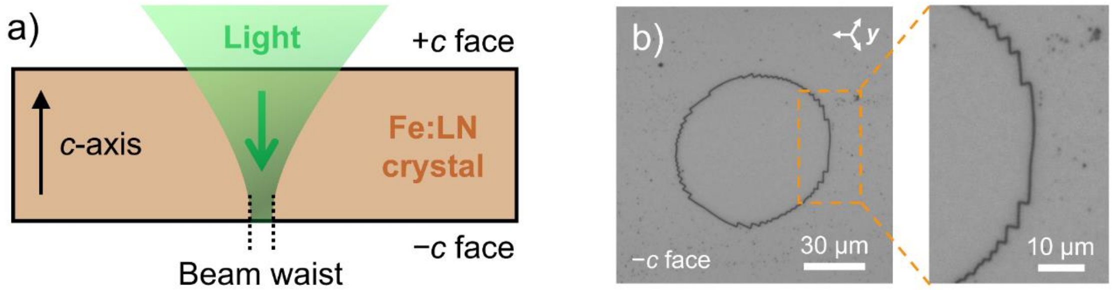

Figure S11. a) Schematic diagram of the focusing conditions employed in Figure 5, with the laser beam waist at the bottom face (the −*c* face). b) Example of a quasi-circular domain obtained with these focusing conditions, using an intensity of 0.42 MW cm^{-2} and an exposure time of 9 seconds. The inset shows how the domain wall is made of multiple sharp steps, with the straight facets preferentially perpendicular to the *y* directions. The location of the longest straight facets coincides with the −*y* sides of the circle.

For further characterization, we also made additional Čerenkov SHG measurements, illustrated in Figure S12. In Figure S12a, the bulk domain structure is somewhat different with respect to Figure 4. In this case, the in-plane geometry of the "spike" domains is not triangular but irregular, and mainly elongated along *y* directions. However, all other relevant features are analogous. Namely, the outer part of the circular domain can grow deeper along the *c*-axis than the central part, giving rise to a ring of domains at the edge that becomes thinner along *z* until it disappears. In this configuration with a focused laser beam, we have measured a remarkable maximum depth of 289 ± 9 μm (see Figure S12b). Moreover, even deeper domains can probably be attained by using higher intensities and longer exposure times. On the other hand, analogously to Figure 4, we have found that exposure alone does not determine the maximum depth of the domains (see Figure S12c), unlike the lateral growth at the surface (see Figure 5).

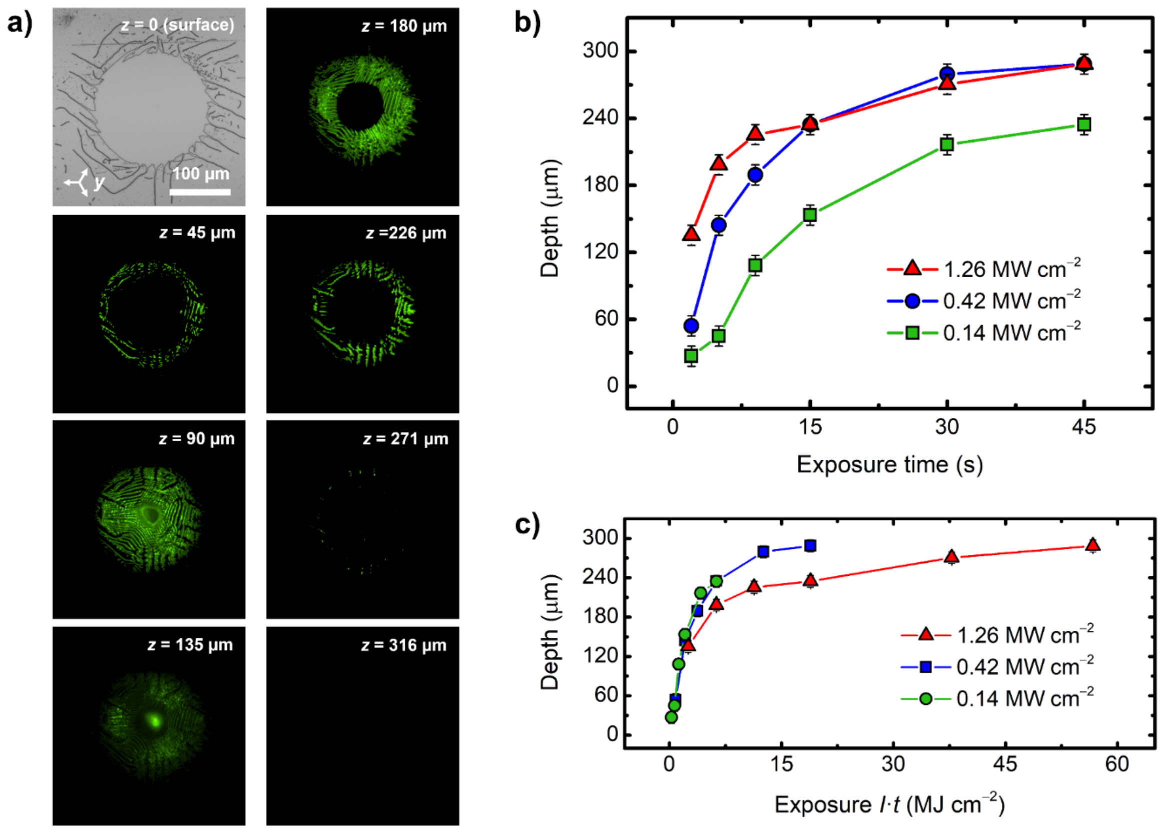

Figure S12. a) Representative measurements of one of the inverted spots of Figure 5 (I = 1.26 MW cm^{-2}, t = 30 s) at different depths using a Čerenkov SHG microscope. The image for z = 0 corresponds to etching at the surface. Green areas indicate the SHG signal coming from domain walls. The graphs show the maximum depth reached by the domains as a function of b) exposure time and c) exposure. The solid lines have been plotted as a guide for the eye.
